# Salicylic Acid Induces Resistance in Rubber Tree against *Phytophthora palmivora*

**DOI:** 10.3390/ijms19071883

**Published:** 2018-06-26

**Authors:** Nuramalee Deenamo, Arnannit Kuyyogsuy, Khemmikar Khompatara, Thitikorn Chanwun, Kitiya Ekchaweng, Nunta Churngchow

**Affiliations:** 1 Department of Biochemistry, Faculty of Science, Prince of Songkla University, Hat-Yai, Songkhla 90112, Thailand; nuramalee.dee@gmail.com (N.D.); kit_kitiya@hotmail.com (K.E.); 2 Department of Chemistry, Faculty of Science and Technology, Nakhon Si Thammarat Rajabhat University, Nakhon Si Thammarat 80280, Thailand; arnannit.k@gmail.com; 3 Office of Agricultural Research and Development Region 8, Department of Agriculture, Ministry of Agriculture and Cooperatives, Hat-Yai, Songkhla 90110, Thailand; kjoy2000@hotmail.com; 4 Faculty of Science and Technology, Rajamangala University of Technology Srivijaya Nakhon Si Thammarat Saiyai Campus, Thungsong, Nakhon Si Thammarat 80110, Thailand; jubthiti@hotmail.com

**Keywords:** salicylic acid (SA), disease resistance, catalase (CAT), phenylalanine ammonia lyase (PAL), defense responses, rubber tree

## Abstract

Induced resistance by elicitors is considered to be an eco-friendly strategy to stimulate plant defense against pathogen attack. In this study, we elucidated the effect of salicylic acid (SA) on induced resistance in rubber tree against *Phytophthora palmivora* and evaluated the possible defense mechanisms that were involved. For SA pretreatment, rubber tree exhibited a significant reduction in disease severity by 41%. Consistent with the occurrence of induced resistance, the pronounced increase in H_2_O_2_ level, catalase (CAT) and peroxidase (POD) activities were observed. For defense reactions, exogenous SA promoted the increases of H_2_O_2_, CAT, POD and phenylalanine ammonia lyase (PAL) activities, including lignin, endogenous SA and scopoletin (Scp) contents. However, SA had different effects on the activity of each CAT isoform in the particular rubber tree organs. Besides, three partial cDNAs encoding CAT (*HbCAT1*, *HbCAT2* and *HbCAT3*) and a partial cDNA encoding PAL (*HbPAL*) were isolated from rubber tree. Moreover, the expressions of *HbCAT1*, *HbPAL* and *HbPR1* were induced by SA. Our findings suggested that, upon SA priming, the elevated H_2_O_2_, CAT, POD and PAL activities, lignin, endogenous SA and Scp contents, including the up-regulated *HbCAT1*, *HbPAL* and *HbPR1* expressions could potentiate the resistance in rubber tree against *P. palmivora*.

## 1. Introduction

Rubber tree (*Hevea brasiliensis* Muell. Arg.) is one of the economically important crops in Thailand. Its products are currently exported worldwide and produce major revenue for the country. *Phytophthora palmivora*, an aggressive hemibiotrophic oomycete pathogen, is a causal agent of abnormal leaf fall and black stripe diseases of the rubber tree. It infects petioles causing extensive defoliation of the mature leaves within a few weeks and attacks tapping surfaces leading to dark vertical lines in the panels, thereby reducing plant growth and latex productivity, subsequently leading to significant economic loss [1].

Upon sensing of the invading pathogens, plants have evolved multitudinous constitutive and induced basal defense mechanisms to protect themselves against pathogen attack, for examples, oxidative bursts, defense signaling pathways, transcriptional expressions of pathogenesis-related (PR) genes, the accumulations of antimicrobial proteins; phytoalexins, and the depositions of lignin, suberin and callose to reinforce cell wall at the pathogen penetration site [2].

Oxidative burst is generally defined as a rapid and transient production of huge amounts of reactive oxygen species (ROS), which is one of the earliest cellular responses to successful pathogen recognition. This initial reaction is the prerequisite for the stimulation of the signaling network that involves in orchestrating the hypersensitive response (HR) and the onset of overall defense responses [3].

The production of ROS is an inevitable effect of the oxidative cellular metabolism. In plants, ROS are regularly synthesized as by-products of photosynthesis, photorespiration, β-oxidation of fatty acids and electron transport in chloroplasts, mitochondria, and endoplasmic reticulum [4]. ROS include free radicals, such as superoxide radical (^•^O_2_^−^), hydroxyl radical (^•^OH), as well as, non-radical molecules, such as singlet oxygen (^1^O_2_) and hydrogen peroxide (H_2_O_2_) and so forth [5]. Among ROS species, H_2_O_2_ is the most relatively stable non-radical ROS and able to diffuse through aquaporins across cell membranes and reaches cell locations distant from its primary generation sites [6]. It is, therefore, considered acting as a signal molecule in the regulation of a variety of biological processes during normal physiological and stressful conditions [7]. The elevated level of H_2_O_2_ during the invasion by pathogens not only serves as a direct protectant against pathogen infection, but also is an intercellular of an intracellular signaling molecule in the induction of systemic acquired resistance (SAR) and exerts the orchestration of hypersensitive cell death at the site of infection [8]. Normally, plants possess the ability to control the ROS toxicity and utilize ROS as signal molecules for initiating defense mechanisms to maintain ROS homeostasis by various ROS-detoxifying systems, including enzymatic and non-enzymatic antioxidant components [9]. Catalase (CAT), ascorbate peroxidase (APX) and guaiacol peroxidase (POD) are H_2_O_2_-scavenging enzymes that play a crucial role in the suppression of toxic H_2_O_2_ levels with different mechanisms in plants [10]. CAT is considered to be more effective for H_2_O_2_ detoxification as it can decompose H_2_O_2_ without consuming cellular reducing equivalents, while APX needs a reduced form of ascorbate and POD requires a phenolic compound guaiacol as an electron donor to eliminate H_2_O_2_. On the contrary, CAT has a lower affinity for H_2_O_2_ (mM range) than APX (μM range) [11].

CAT (EC 1.11.1.6) is a heme-containing homotetrameric enzyme that catalyzes H_2_O_2_ to water and oxygen and abundantly localizes to peroxisomes which is also found in cytosol, mitochondria and chloroplast [12]. Plant CAT exists in multiple forms encoded by members of a small gene family. Each CAT isoenzyme exhibits a unique expression to developmental and environmental stimuli. It has been reported that tobacco, maize, rice, pumpkin and Arabidopsis contained three *CAT* genes encoding to different types of enzyme CAT [13].

POD (EC 1.11.1.7), a heme-containing protein, is an oxidoreductase enzyme that catalyzes the oxidation of many organic and inorganic substrates using H_2_O_2_ as the electron acceptor [14]. POD is primarily localized in vacuoles, cell wall and cytosol [15]. POD encoded by a large multigene family that comprises several isoenzymes with putative functional specialization [16]. POD is present in wide substrate specificities. Lacking strict substrate specificity suggests that POD is associated with various physiological processes in plants, such as the metabolism of auxin, cell wall modification (e.g., lignification, and suberinization), the biosynthesis of phenolic compounds and defense against pathogens [17].

Besides, plants often respond to pathogens by producing specific phenolic substances, which play a crucial role in pre-invasive structural defenses including the reinforcement of cell walls (e.g., lignin and callose depositions), and productions of toxic compounds (e.g., phytoalexins) at attempted penetration sites [18].

Phenylalanine ammonia lyase (PAL; EC 4.3.1.24) is the first and committed step in the phenylpropanoid pathway that catalyzes the non-oxidative deamination of phenylalanine to trans-cinnamic acid and ammonia [19]. Trans-cinnamic acid is consequently a precursor for the biosynthesis of various polyphenyl compounds such as lignins, flavonoids, plant hormones (e.g., SA), and phytoalexins (e.g., scopoletin; Scp), which are involved in pigmentation, growth, reproduction, plant defenses and many other functions [20,21,22]. In many plant species, PAL exists in many isoforms encoding by a multigene family [23,24]. The PAL activity can be induced in response to developmental processes and various stress conditions, including wounding, pathogenic attack, UV light, and phytohormones such as SA [25].

To date, the use of chemical fungicides in disease management is the most effective tool. However, repeated use has led to serious problems, such as environmental pollution, fungicide resistance, and residual toxicity. Exploiting uniquely the plant potential to fight pathogens, the induced resistance by natural or synthetic compounds may minimize the use of fungicides for disease control, and therefore could be considered as an alternative and environment-friendly approach for plant protection in the sustainable agricultural production.

Salicylic acid (SA), an endogenous elicitor, is an important defense hormone mediating in signal transduction systems, which can stimulate both localized acquired resistance (LAR) and systemic acquired resistance (SAR) [26]. SA is predominantly correlated with resistance against biotrophic and hemibiotrophic pathogen [27]. Exogenous application of SA results in resistance against certain pathogens, which is related not only to the induction of SAR and expressions of PR-genes [28], but also potentiated to respond abruptly and efficiently with diverse defensive strategies against subsequent challenge by pathogens [29].

The present study was conducted to investigate the effect of exogenous SA on the accumulation of H_2_O_2_ level, the activities of defense-related enzymes (CAT, POD and PAL), lignin deposition, amounts of total phenolic compounds, endogenous SA and Scp; also analyzed the expressions of *HbCAT1*, *HbPAL* and *HbPR1*, since these components might function synergistically in priming defenses in rubber tree against subsequent invasion by *P. palmivora*.

## 2. Results

### 2.1. Effects of SA Priming on Resistance of Rubber Tree

#### 2.1.1. Induced Resistance of SA-Pretreated Rubber Tree against *P. palmivora*

**Figure 1:**
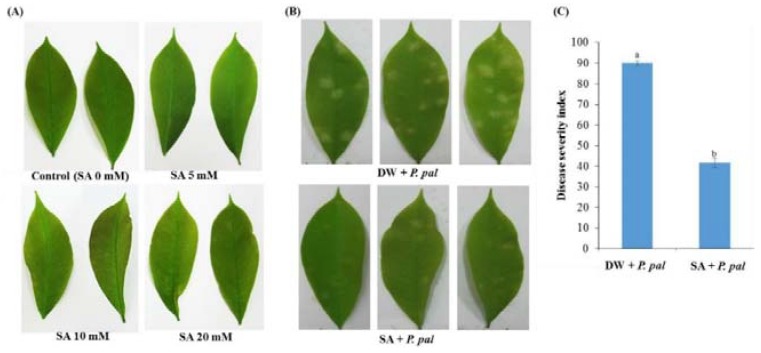
Effect of exogenous SA treatment on rubber tree against *P. palmivora.* (**A**) Physio-morphological traits of rubber tree leaves at 1 day after treatment with SA at different concentrations of 0, 5, 10 and 20 mM; (**B**) Disease symptom; and (**C**) disease severity (%) of rubber tree leaves pretreated with either DW or 5 mM SA for 1 day prior to subsequent inoculation with *P. palmivora* zoospore suspensions (1 × 10^5^ zoospores mL^−1^) at 4 dpi. The columns and vertical bars represent means ± standard errors (SE) of three independent replicates of 10 seedlings. Pretreated plants and non-pretreated control differ significantly at *p* ≤ 0.05, according to paired-samples *t*-test. All leaves were detached from the seedlings at 4 dpi for better pictorial representation.

In this study, we investigated the effect of SA priming on induced resistance in rubber tree against *P. palmivora* infection. In our preliminary study, the effect of SA on the leaf of rubber tree seedlings was tested with various concentrations (0, 5, 10 and 20 mM). After 1 day of the treatment, the leaves of some SA-treated plants at 10 mM and 20 mM showed leaf shrinkage, while there was no shrinking effect on leaves of SA treated with 5 mM (Figure 1A). Henceforth, we selected 5 mM SA for further experiments.

The exogenous 5 mM SA could induce resistance in rubber tree when compared to the control (Figure 1B,C). Leaf symptoms were evaluated at 0, 1, 2, 3 and 4 dpi, the increased necrotic lesion numbers and the rapidly expanded *P. palmivora* growth were obviously revealed on the infected rubber tree leaves in contrast to its disease symptom in the SA pretreatment with 5 mM which showed the remarkably decreased necrotic lesion numbers and disease severity by 41.67% (Figure 1B,C). The results indicated that SA pretreatment could effectively inhibit *P. palmivora* growth on rubber tree leaves.

#### 2.1.2. Effect of SA Priming on H_2_O_2_ Content, Total Protein Content, CAT and POD Activities and Total Phenolic Content in Rubber Tree Leaves after *P. palmivora* Challenge

**Figure 2:**
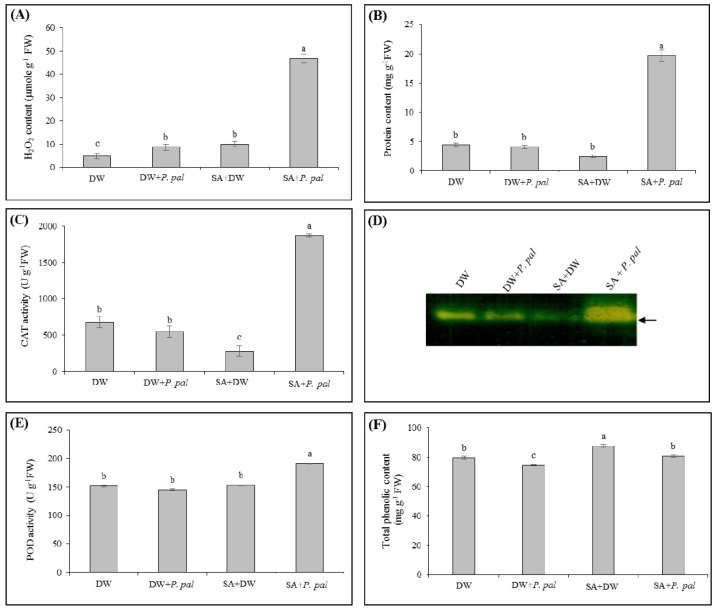
Effect of exogenous SA pretreatment on H_2_O_2_ content, total protein content, CAT and POD activities and total phenolic content in rubber tree leaves after inoculation with *P. palmivora* (*P. pal*). The leaves of rubber tree seedlings were sprayed with either distilled water (DW) or 5 mM SA. After 1 day of the treatment, leaves were subsequently treated with either DW or *P. palmivora* zoospore suspensions at 1 × 10^5^ zoospores mL^−1^. After 4 dpi, the leaf samples were collected for determining (**A**) H_2_O_2_ content; (**B**) total protein content; (**C**) CAT activity; (**D**) CAT activity staining; (**E**) POD activity; and (**F**) total phenolic content. The columns and vertical bars represent mean ± standard errors (SE) of three independent replicates of 10 seedlings. Different letters represent significant differences, according to Tukey’s HSD test at *p ≤* 0.05. Arrows indicate the position of CAT activity band.

Priming rubber tree seedlings with 5 mM SA before *P. palmivora* inoculation (SA + *P. pal*) caused a significant increase in H_2_O_2_ content by 9.44-fold, total protein content by 4.45-fold and CAT activity by 2.76-fold, whereas inoculation with *P. palmivora* (DW + *P. pal*) or treatment with 5 mM SA (SA + DW) slightly affected their levels, as compared to the control (Figure 2A–C). In addition, the activity band, visualized by native-PAGE, showed patterns similar to that of CAT activity determined by spectrophotometry (Figure 2C,D).

For POD, the activity was not significantly increased after triggering with *P. palmivora* or SA. However, in the leaves treated with 5 mM SA prior to *P. palmivora* inoculation, the POD activity was increased by 1.26-fold when compared to the control (Figure 2E).

Differently, the treatment with SA (SA + DW) resulted in an increase of total phenolic content by 1.10-fold in rubber tree leaves as compared to the control (DW) (Figure 2F). In the leaves that were treated with SA before *P. palmivora* inoculation, total phenolic content was higher than that of the inoculation with *P. palmivora* (DW + *P. pal*) even though it was not significantly different from the control (DW).

### 2.2. Effect of SA on Kinetics of H_2_O_2_ Content, CAT and PAL Activities, SA and Scp Contents in Rubber Tree Seedling Leaves

#### 2.2.1. Effect of SA on H_2_O_2_ Content

**Figure 3:**
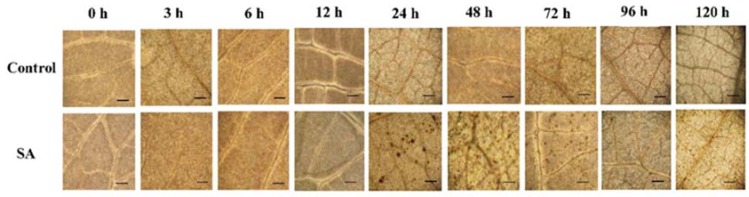
In situ detection of H_2_O_2_ using 3,3′-diaminobenzidine (DAB) staining on rubber tree leaves after treatment with 5 mM SA. The rubber tree leaves were sprayed with either DW as a control or 5 mM SA. Leaf pieces were collected and stained after treatment at different time points (0, 3, 6, 12, 24, 48, 72, 96 and 120 h). Pictures represent three independent biological replicates. The dark brown precipitates indicate the presence and distribution of H_2_O_2_ in plant cells, which could be visualized using a light microscope (scale bars = 100 μm).

To determine the effect of SA on the induction of H_2_O_2_ content in rubber tree, we used DAB staining method to detect the distribution of H_2_O_2_ in rubber tree leaf cells. The results revealed that the SA treatment caused a great accumulation of H_2_O_2_ in leaf cells at 24 h till 72 h, predominantly along the veins appearing as dark brown spots. Whereas, the control leaves showed a low level of H_2_O_2_ as appearing a weak background of DAB staining (Figure 3). The results indicated that SA could induce an increase in the rate of generation of H_2_O_2_ in rubber tree leaves.

#### 2.2.2. Effect of SA on CAT, POD and PAL Activities

**Figure 4:**
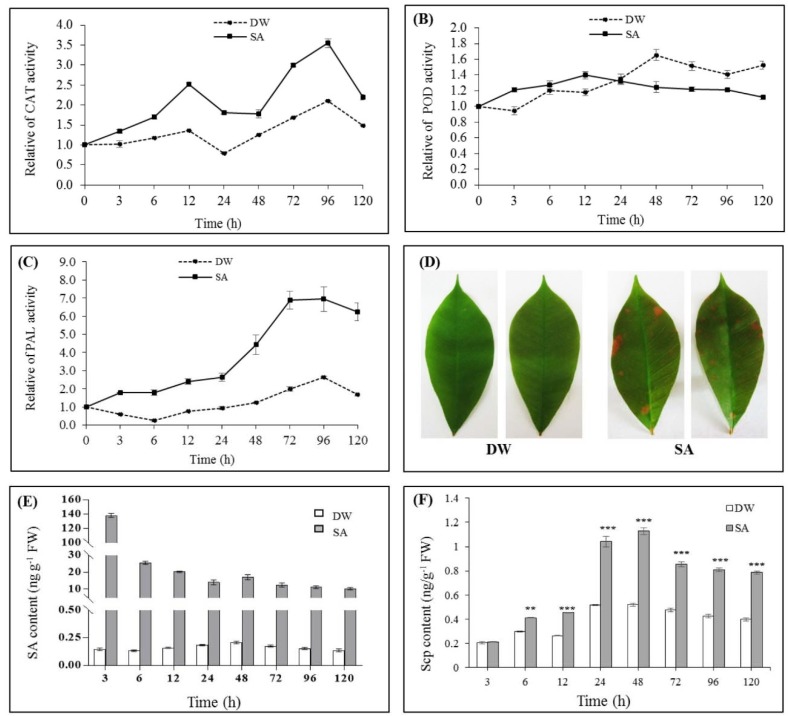
The effect of SA on (**A**) CAT; (**B**) POD; (**C**) PAL activities; (**D**) lignin; (**E**) endogenous SA; and (**F**) Scp contents in rubber tree. The leaves of rubber tree seedlings were sprayed with either distilled water (DW) as control or 5 mM SA and harvested at different points of time (3, 6, 12, 24, 48, 72, 96 and 120 h) for enzyme activity measurements by spectrophotometry and endogenous SA and Scp detections by HPLC. All data represent mean ± SE of three biological replicates of 10 seedlings. Asterisks indicate statistically significant differences in the level of Scp content in SA treatment compared to its content in the control at the same time point (** for *p* ≤ 0.005, *** for *p* ≤ 0.001), according to paired-samples *t*-test. Lignin deposition was visualized by phloroglucinol-HCl staining of the rubber tree leaves treated with 5 mM SA at 48 h.

The effect of SA on the activities of CAT, POD and PAL was investigated in rubber tree leaves after treatment with 5 mM SA over a time-course. The results showed that CAT activity was continuously increased at 3–6 h, culminated at 12 h (1.85 fold) and slightly decreased at 24–48 h, then increased at 72 h and peaked again at 96 h (1.69 fold) when compared to the control, and abruptly declined thereafter (Figure 4A).

For POD, the activity was slightly increased from 3–12 h, subsequently remained at the same level of the control at 24 h and then decreased to a lower level than the control at 48–120 h (Figure 4B).

For PAL, the activity was continuously increased at 3 h till 48 h and reached the highest level at 72 h (3.48 fold) when compared to the control and started to decline from 96–120 h (Figure 4C).

#### 2.2.3. Effect of SA on Lignin Deposition

After rubber tree seedlings were treated with either distilled water (DW) or 5 mM SA for 48 h, the leaves of seedlings were harvested and stained with phloroglucinol-HCl to detect lignin deposition. The results indicated that SA-treated rubber tree leaves revealed an increase in lignin, appearing as red spots, while the control ones were slightly stained (Figure 4D). The results suggested that SA could induce lignin deposition in rubber tree leaves.

#### 2.2.4. Effect of SA on Endogenous SA and Scp Levels

To determine whether exogenous SA application could induce the biosynthesis of endogenous SA and Scp in rubber tree, we sprayed 5 mM SA on the leaves of rubber tree seedlings and collected the treated leaves at different times. All leaves were washed thoroughly three times with distilled water and dried with absorbent paper to remove the remaining SA on the leaf surface before analysis. The results showed that extremely high content of endogenous SA was detected in the SA-treated leaves at 3 h (138.11 ng g^−1^ FW). The obtained high value of SA content in the leaf might be due to some penetration of exogenous SA at the early time. In the SA-treated leaves, the endogenous SA content was diminished continuously at 6–24 h, slightly increased at 48 h (17.09 ng g^−1^ FW) and then decreased at 72 h till 120 h. In contrast, the endogenous SA content was barely detected in the control leaves (less than 0.21 ng g^−1^ FW) throughout the experimental period of 120 h (Figure 4E).

Furthermore, the Scp content in the SA-treated leaves was found to be increased continuously at 6–24 h and reached the highest content at 48 h (1.13 ng g^−1^ FW), and declined thereafter (Figure 4F). Interestingly, a prominent increase in Scp content at 48 h was correlated with the increased endogenous SA in the SA-treated leaves (Figure 4E,F). The results indicated that exogenous SA could induce the biosyntheses of endogenous SA and Scp in rubber tree.

### 2.3. Effect of SA on CAT Activity in Different Rubber Tree Organs

To determine the effect of SA on CAT activity in different rubber tree organs, crude protein was extracted from the leaves, stems, hypocotyls and roots of in-vitro rubber tree plantlets grown in MS medium supplemented with 5 mM SA and subjected to determine CAT activity.

**Figure 5:**
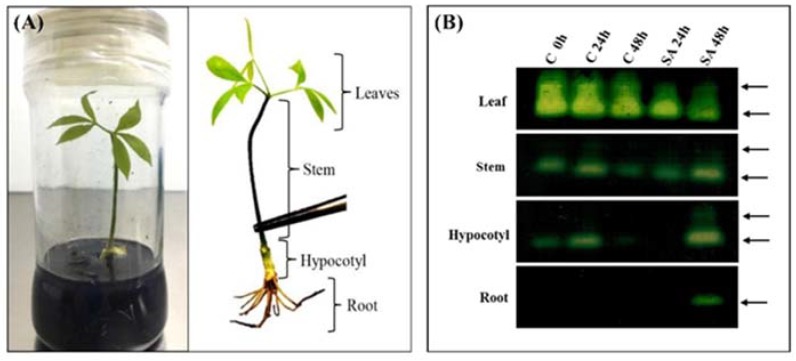
Effect of SA on CAT activity in different rubber tree organs. (**A**) The 45-day-old in-vitro culture of rubber tree plantlets grown in MS medium supplemented with 5 mM SA; and (**B**) The CAT activity staining after native-PAGE of crude protein from each organ (leaves, stems, hypocotyls and roots) of rubber tree plantlets treated with 5 mM SA at indicated times (0 h, 24 h and 48 h), equal volume of crude extract (5 μL) of each rubber tree organ was loaded on native-PAGE and stained for CAT activity. Arrows indicate the position of CAT activity band.

The CAT activity was highly found in the leaves than stems and hypocotyls, respectively and barely detected in the roots of in-vitro plantlets. After treatment with SA, the CAT activity in the leaves started to decrease at 24 h and decreased even more at 48 h. On the other hand, the CAT activity in the stems, hypocotyls and roots turned to remarkably increased at 48 h. The results suggested that SA led to different changes in CAT activity of each organ of rubber tree plantlets (Figure 5B).

### 2.4. Cloning and Sequence Analysis of Three Partial cDNAs Encoding Catalases

**Figure 6:**
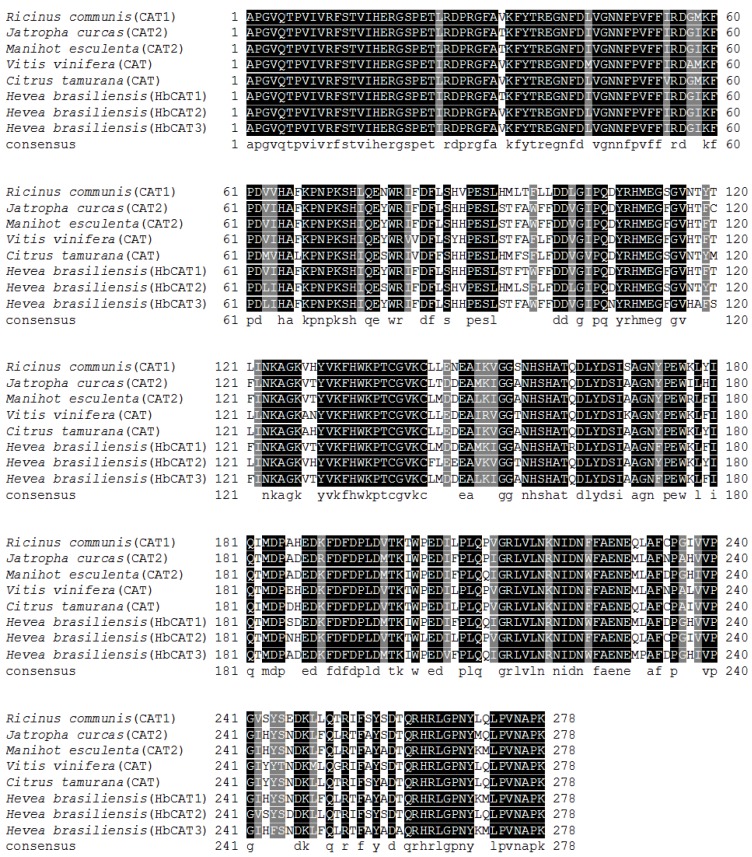
Multiple alignment of the deduced amino acid of HbCAT1, HbCAT2 and HbCAT3 with catalases from other plants, *R. communis* (BAA04697), *J. curcas* (NP_00129295.1), *M. esculenta* (AAD50974.1), *V. vinifera* (NP_001268098.1) and *C. tamurana* (BAQ21275.1). The conserved amino acid residues are shaded dark and the highly conserved amino residues are shaded grey.

By RT-PCR amplification, three 834-bp nucleotide sequences were obtained from total RNA samples of the leaf and the root of in-vitro rubber tree plantlets. All nucleotide sequences encoding 278 amino acid residues showed similarities to other plant catalases (Figure 6). Two sequences obtained from the leaf were named as *H. brasiliensis catalase 1* (*HbCAT1*) and *H. brasiliensis catalase 2* (*HbCAT2*) and another one obtained from the root was named as *H. brasiliensis catalase 3* (*HbCAT3*). Three partial cDNA sequences of *HbCAT1*, *HbCAT2* and *HbCAT3* have been deposited in the National Center of Biotechnology Information GenBank (NCBI) under the accession no. MF383167, MH010572 and MH010573, respectively. The amino acid sequences of HbCAT1, HbCAT2 and HbCAT3 are shown in Figure 6.

A GenBank BlastP comparison revealed that HbCAT1 exhibited a high similarity with *Manihot esculenta* catalase 2 at 97% (XP_021601170.1), *Jatropha curcas* catalase 2 at 94% (NP_001292952.1) and *Ricinus communis* catalase at 91% (EEF52864.1), respectively. The HbCAT2 showed a high similarity with *M. esculenta* catalase 1 at 96% (XP_021612700.1), *J. curcas* catalase 1 at 95% (XP_012073648.1) and *R. communis* catalase at 91% (EEF52864.1), respectively. The HbCAT3 showed a high similarity with *M. esculenta* catalase 2 at 95% (XP_021601170.1), *J. curcas* catalase 2 at 91% (NP_001292952.1) and *R. communis* catalase 2 at 88% (XP_015574606.1), respectively (Figure 6).

### 2.5. Cloning and Sequence Analysis of a Partial cDNA Encoding Phenylalanine Ammonia Lyase

**Figure 7:**
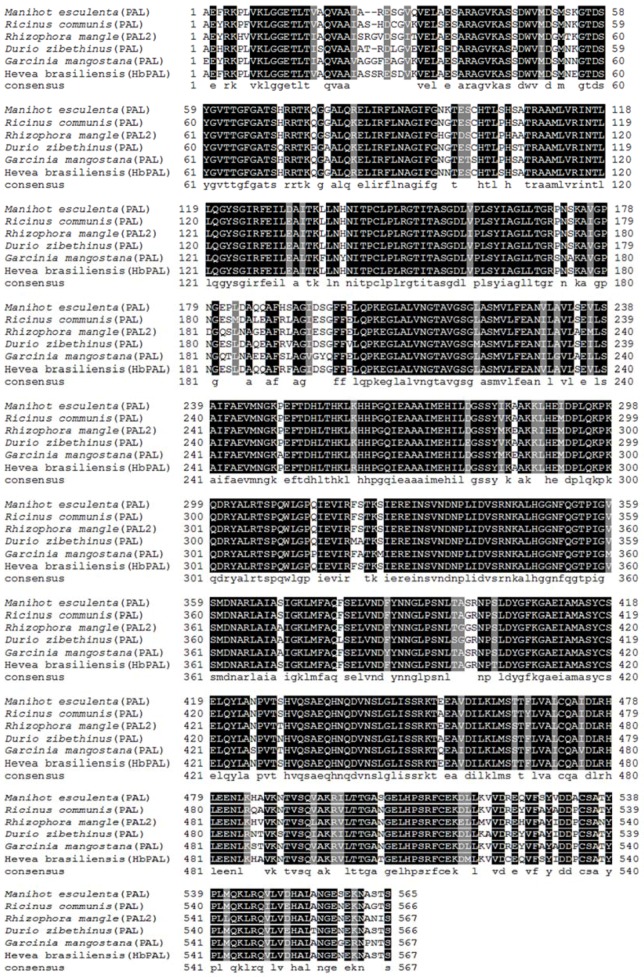
Multiple alignment of the deduced amino acid sequences of HbPAL with phenylalanine ammonia lyase from various plants, *M. esculenta* (XP_021620397.1), *R. communis* (XP_002519521.1), *R. mangle* (AAW51923.2), *D. zibethinus* (XP_022744606.1) and *G. mangostana* (ACM62741.1). The conserved amino acid residues are shaded dark and the highly conserved amino residues are shaded grey.

A 1701 bp nucleotide sequence was obtained from total RNA of rubber tree seedling leaves. This sequence, named as *HbPAL*, exhibited similarities to the *PAL* genes of other plants. The partial cDNA of *HbPAL* has been deposited in NCBI under the accession no. MG992015. The deduced HbPAL amino acid sequences (567 and amino acid residues) showed a high similarity with *M. esculenta* phenylalanine ammonia lyase 2 at 96% (AAK60275.1), *J. curcas* phenylalanine ammonia lyase at 94% (XP_012082374.1), and *R. communis* phenylalanine ammonia lyase at 93% (AGY49231.1) (Figure 7).

### 2.6. Effect of SA on HbCAT1, HbPAL and HbPR1 Expressions in H. brasiliensis

**Figure 8:**
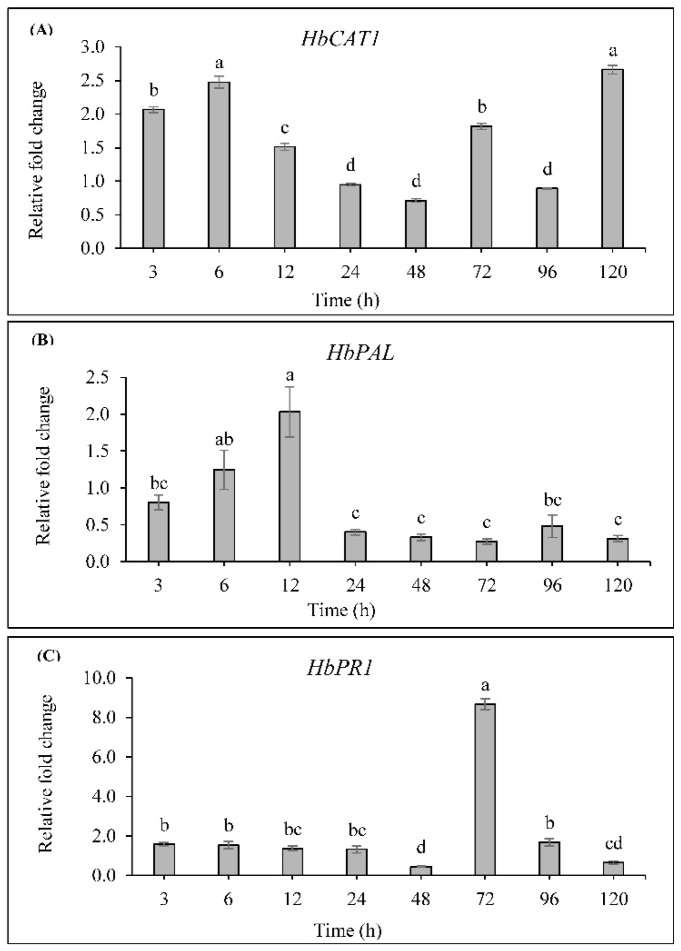
Effect of SA on transcript abundant of (**A**) *HbCAT1*; (**B**) *HbPAL*; (**C**) *HbPR1* genes in rubber tree. The leaves of rubber tree seedlings were sprayed with either distilled water or 5 mM SA. Total RNA was extracted from the leaves taken at various time points (3, 6, 12, 24, 48, 72, 96 and 120 h), converted to cDNA, and subjected to semi-qRT-PCR. Relative transcript levels were calculated relative to expression of *Hbmitosis* mRNA. The expression levels of *HbCAT1*, *HbPAL* and *HbPR1* genes were expressed as a relative transcript fold change to their controls. All data show the average of three replications. Error bars indicate standard errors. Different letters represent significant differences, according to Tukey’s HSD test at *p* ≤ 0.05.

To investigate the effect of SA on rubber tree defense gene expression, the expressions of *HbCAT1*, *HbPAL* and *HbPR1* were determined by semi-qRT-PCR. The expression of *HbCAT1* was induced by 2.07-fold and 2.48-fold at 3 h and 6 h, respectively, in the SA-treated plants, then the expression decreased and was suppressed at 48 h when compared to the control plants. Subsequently, the expression was induced by 1.82-fold and 2.66-fold at 72 h and 120 h, respectively (Figure 8A).

For *HbPAL*, the expression was slightly up-regulated at 6 h and reached the highest level at 12 h with 2.03-fold of control, thereafter the expression was suppressed to below the level of control (Figure 8B).

The expression of *HbPR1* was slightly induced at 3 h till 24 h and greatly induced by 8.67-fold of control at 72 h, then the expression was rapidly declined at 96 h and suppressed to below the level of control at 120 h (0.65-fold) (Figure 8C).

## 3. Discussion

*P. palmivora* is one of the significant destructive plant pathogens that severely threaten rubber tree cultivation and latex production [1]. Traditional methods for disease control depend mainly on the application of chemical fungicides; however, the use of them has various hazardous effects on the environment and human health, including the emergence of highly resistance fungal strains. Induced resistance exploiting intrinsic defense mechanisms of plants by specific biotic or abiotic elicitors is proposed as an eco-friendly approach to protect plants against disease. Its introduction into agricultural practice may reduce chemical applications, therefore contributing to the development of sustainable agriculture [30].

SA has long been recognized to play a central role in the regulation of priming defense responses against various pathogenic infections [31] and induces SAR in plants [32]. Several studies have supported that the application of SA can induce resistance to various types of pathogens [26,27,30]. Pretreatment with SA primes the plant cells to react more rapidly and effectively to subsequent pathogen attack [33].

In this study, we found that the pretreatment of 5 mM SA provided significant protection in rubber tree against *P. palmivora* infection. The biochemical changes of rubber tree treated either with SA alone or SA prior to subsequent inoculation with *P. palmivora* were then investigated. For priming study, SA could reduce necrotic lesion numbers and mitigate the expansion of *P. palmivora* mycelium on the infected leaves. In addition, the resistance stimulating effects of this study lasted for at least 5 days in rubber tree leaves (Figure 1). Our finding was similar to that of Zhang et al. (2016) who reported that SA could induce resistance in ‘Gala’ apple leaves against *Glomerella cingulata* [30]. Moreover, Mandal et al., (2009) reported that the application of SA reduced the disease severity and conferred resistance to *Fusarium oxysporum* f. sp. *lycopersici* in tomato [32].

One of the earliest responses of plant-pathogen interactions is the rapid accumulation of ROS at the pathogen attack site (a phenomenon called oxidative burst), which plays an important role in the plant immune system [34]. These ROS can destroy invading pathogens directly and participated in the orchestration of hypersensitive response (HR). H_2_O_2_ is a stable intermediate of ROS and acts as a diffusible selective signal for inducing the expression of genes encoding proteins involved in defensive and antioxidant processes [35].

After rubber tree seedlings were primed with SA for 1 day then challenged by *P. palmivora* for 4 days, the level of H_2_O_2_ and the activities of CAT and POD (SA + *P. pal* of Figure 2A,C–E) were abundantly increased when compared to the unprimed plants (DW, DW + *P. pal*, SA + DW of Figure 2A,C–E). The predominant H_2_O_2_ after SA pretreatment might participate in the induced resistance in rubber tree via direct killing the invading pathogen and/or stimulating subsequent defense response. According to the result of Mejía-Tenieute et al. (2013), exogenous application of SA resulted in the induction of endogenous H_2_O_2_ level and increased of PAL and CAT activities, as well as, the activation of defense-related gene expressions (*CAT1*, *PAL* and *PR1*) which possibly triggered induced resistance to biotic stress in *Capsicum annuum* L. [36]. However, an excess H_2_O_2_ level is also harmful to plant cells and must simultaneously be detoxified by antioxidant enzymes, such as CAT and POD [2]. Our data showed that the activity of CAT was enhanced much higher than that of POD in rubber tree pretreated with SA before inoculation with *P. palmivora* (Figure 2C–E), which have resulted from the degrading function of CAT at relatively high H_2_O_2_ concentration. The massive accumulation of H_2_O_2_ during the pathogen-induced oxidative burst caused less damage and that might be due to the induction of these detoxifying enzyme activities in rubber tree.

In addition to detoxifying function, POD is one of the key regulatory enzymes for the biosynthesis of a variety of secondary metabolites in plants [37]. It is well known that plant secondary metabolites are involved in plant defense responses against pathogens and herbivores [38]. Our current study also showed the increase of total phenolic content in the rubber trees treated with SA before inoculated with *P. palmivora* (SA + *P. pal* of Figure 2F) when compared to the unprimed plants (DW + *P. pal* of Figure 2F). We speculate that the SA pretreatment caused a consequent chain of defense responses and subsequently resulted in the induced resistance in rubber tree against *P. palmivora* infection. Similarly, the previous study in tomato plants proposed that the SA pretreatment decreased the percent of vascular browning and plant wilting, leading to the reduction of bacterial wilt disease incidence and induced total phenolic compounds and defense-related enzymes in tomato leaves when inoculated with *Ralstonia solanacearum* [39].

The effect of SA as abiotic elicitor was also investigated in rubber tree. We found that the exogenous application of SA could induce the accumulation of H_2_O_2_ (Figure 3) and a change in the levels of CAT and POD activities (Figure 4A,B). CAT and POD are considered as main antioxidant systems to protect cells against oxidative damage [40]. It was proven that SA increases the activities of CAT, POD and PAL, as well as, the defensive compounds [20,41,42]. Our results also revealed a significant increase in activity of PAL with a concomitant increase in lignin content, including endogenous SA and Scp (Figure 4C–F). Enhancement of POD, PAL and lignin in SA-treated rubber tree might result in the reinforcement of cell wall and formation of a physical barrier to restrict the penetration of pathogen [42]. These results are supported by Mandal (2010), who suggested that SA acted as elicitor in inducing POD and PAL activities and lignin deposition leading to cell wall strengthening in eggplant roots [43]. Scp is a phytoalexin found in rubber tree which showed an effective fungitoxicity to inhibit the mycelium growth of *P. palmivora* and other pathogens [44]. In addition, Dorey et al. (1999) reported that the production of H_2_O_2_ from the oxidative burst could elicit activity of PAL and consequent stimulation of endogenous SA and Scp in cultured tomato cells [45].

Even though, there are many pieces of evidences that SA increased the activity of CAT [30,45,46], its activity in rice, wheat and cucumber was decreased after SA treatment [47]. We found two peaks of CAT activity after SA treatment; however, CAT activity was decreased at 24 h and 48 h (Figure 4A) while endogenous SA was detected at 48 h (Figure 4E). Furthermore, it has been proposed that SA could bind to iron-containing enzymes (SA binding proteins including CAT), resulting in the inhibition of CAT activity which might be due to its conformation change [48]. It was suggested that the reduction of CAT activity would maintain the level of H_2_O_2_ for signaling propose in plant defense [49]. Corresponding to our results, previous studies reported that SA caused the increase of H_2_O_2_ level, while the decline of CAT activity was occurred [50,51]. For POD kinetics, our study showed that the activity was decreased at 24–120 h (Figure 4B). The reduction of POD in our results and other plants [11,50] might be due to the binding of SA to heme enzymes including POD [48].

In this work, CAT isozymes activities were detected in different organs of in-vitro rubber tree plantlets. We found that the activities of CAT isozymes in the rubber tree leaves were inhibited by SA, whereas the induction of CAT isozyme activities in the stems, hypocotyls and roots were observed. Our findings suggested that SA could result in different regulations of individual CAT isozyme activities (Figure 5B). SA has been proposed to inhibit CAT and thereby enhance the production of H_2_O_2_, which may act as a secondary messenger in plant defenses [52]. In support, Rao et al. (1997) found that SA treatment in *A. thaliana* elevated the H_2_O_2_ content and H_2_O_2_-metabolizing enzymes depended upon the dose of SA concentration and time-duration [50]. In plants, the various CAT present multiple isoforms with different activities in diverse plant organs [53,54,55]. CAT isozymes of chickpea plant were sensitive to SA differently, because SA was a specific inhibitor of CAT activity both in shoot and root [56]. Particularly, inhibition of CAT activity by SA may be involved in SA-mediated induction of SAR in plants [8].

Three partial cDNAs of catalase genes were obtained from rubber tree leaves (*HbCAT1* and *HbCAT2*) and root (*HbCAT3*). The three fragments of *HbCAT* were consisted of 834 bp and encoded 278 amino acid residues. An amino acid sequence of HbCAT1 showed the maximum similarity with catalase 2 of *M. esculenta* (97%) (Figure 6). Drory and Woodson (1992) reported that the nucleotide sequence of *CAT1* from tomato was 1822 bp (492 amino acids) [57]. In this study, we also obtained a fragment of *HbPAL* (1701 bp) which was encoded 567 amino acid residues and showed a high similarity with phenylalanine ammonia lyase 2 of *M. esculenta* (96%) (Figure 7). The full-length cDNA of *PAL* was isolated from *Juglans regia* containing 1935 bp and encoded 645-amino-acid protein [58]. These results indicated that the obtained *HbCAT1* and *HbPAL* were similar to catalase and phenylalanine ammonia lyase, respectively, with other plants.

The response of defense-related genes to exogenously applied SA was examined in rubber tree leaves. Our results showed that the expression of *HbCAT1*, *HbPAL* and *HbPR1* genes was significantly induced by SA (Figure 8A–C). We also observed a significant up-regulation of *HbCAT1* (Figure 8A) which was correlated with the increase of CAT activity (Figure 4A) by SA treatment. Correspondingly, the previous report was shown that catalase genes (*CAT1*, *CAT2*, and *CAT3*) from maize differently responded to exogenous SA application [55]. This event supported the idea that SA may cause oxidative stress via an increase of H_2_O_2_ level [59]; however, pretreatment of plants with the suitable concentrations of SA might induce defense-related genes in scavenging H_2_O_2_ [36]. The increase of three different catalase genes expression in hot pepper and small radish can play an important role in response to environmental stresses [60,61]. In addition to the induction of catalase, the action of SA may have other functions in plants [8].

PAL is considered a key enzyme in plant defense mechanisms since it catalyzes the biosynthesis of various related-defense metabolites including phenolic compounds and lignin. The transcript of *PAL* gene was differentially expressed during plant growth and development [62]. It was required to regulate the activity of PAL as a rate-limiting enzyme in the phenylpropanoid pathway [63]. Silencing of *PAL* genes resulted in the reduction of SA level as PAL enzyme was not accumulated [64]. In addition, previous research reported that SA could activate the expression of *PAL* gene and PAL activity in grape berry [65]. Our results showed a significant induction of *HbPAL* gene (Figure 8B) and PAL activity (Figure 4C) as well as an enhanced amount of lignin, endogenous SA and Scp (Figure 4E,F) in SA-treated rubber tree leaves. It is widely accepted that the accumulation of lignin and Scp involves in supporting the mechanical resistance to pathogen penetration by increasing cell wall reinforcement and potential antimicrobial agent, and therefore forming nonpermeable barriers against pathogens attack [17,21,22].

Moreover, SA is also known to be a natural transduction signal in plant defense response. Application of exogenous SA induced *PR* gene expression and increased resistance to plant disease [66]. An elevated expression of *PR1* gene reported herein (Figure 8C) is consistent with the previous study showing that the expression of *PR1* gene was highly induced in SA treatment of wheat seedling leaves and that correlated with increased resistance during infection of wheat plants by *Blumeria graminis* f. sp. *tritici* [67]. SA treatment induced the production of PR proteins which act as molecular markers for the establishment of defense response [26,67]. Recently, foliar spraying of SA in apple prior to inoculation of *G. cingulata* stimulated the up-regulation of *PR1 gene* and significantly reduced the Glomerella leaf spot disease [30]. Besides, the accumulation of endogenous SA was correlated with the induction of *PR1* gene expression and the activation of SA signaling in defense mechanisms against *Collectotrichum* in strawberry plants [68]. Glazebrook (2001) reported that *NahG*-transgenic plants and *Arabidopsis* mutants impaired in SA production increased the susceptibility to various pathogens and indicated the importance of SA for SAR establishment [69]. In the present investigation, the exogenous SA resulted in the increase of endogenous SA accumulation and the up-regulation of *PR1* gene, probably contributing in increased resistance of rubber tree to *P. palmivora* through the onset of SAR.

From our results and those aforementioned reports, we suggest that the SA-induced *HbCAT1*, *HbPAL* and *HbPR1* gene expressions lead to the biosyntheses of defense-related enzymes and secondary metabolites, thereby inducing resistance in rubber tree against *P. palmivora* infection.

## 4. Materials and Methods

### 4.1. Phytophthora palmivora Culture

*P. palmivora* was routinely grown on potato dextrose agar (PDA) plate at 25 °C. Zoospores were produced from the actively growing *P. palmivora* mycelium on V8 agar, following the method described earlier [70]. In brief, 1-cm agar plugs of *P. palmivora* mycelium from a 7-day culture were transferred to 10% unclarified V8 agar (10% V8 juice, 0.1% CaCO_3_, 1.5% agar) and cultured at 25 °C under fluorescent light for a week. The culture was covered with 10 mL of cold (4 °C) sterile distilled water, incubated at 4 °C for 15 min, and then shaken at 50 rpm for 15 min at room temperature to release the motile zoospores from sporangia. The zoospore concentration was counted using a hemocytometer under light microscope. A concentration of 1 × 10^5^ zoospores mL^−1^ was used for the inoculation of rubber tree leaves.

### 4.2. Plant Materials and Treatments

Twenty-one-day-bud grafted rubber tree (*H. brasiliensis*, RRIM600 cultivar) seedlings were propagated in a climate-controlled room at 25 °C under 12 h/12 h light/dark photoperiod. For treatments, the rubber tree leaves at the developmental B2C stage of plantlets with uniform growth were sprayed with either distilled water (DW) or salicylic acid (SA) at concentrations of 5 mM. After that, the treated seedlings in each treatment group were separately covered with plastic bags to maintain high humidity and incubated in a climate-controlled room. Each treatment was conducted in triplicates and each replicate contained 10 plants. Six leaves were detached from two petioles in the same layer of seedling stem for assays at 0, 3, 6, 12, 24, 48, 72, 96 and 120 h, respectively.

In-vitro culture of rubber tree was carried out, according to the method described earlier [70]. For SA treatment, 45-day-old plantlets were transplanted in the semi-solid Murashige and Skoog’s (MS) [71] medium containing growth hormones supplemented with or without 5 mM SA and maintained in a climate-controlled room at 25 °C, under 12 h-light and 12 h-dark cycle. The leaves, stems, hypocotyls, and roots of in-vitro rubber tree plantlets were collected at each time points (0 h, 24 h and 48 h) of treatment and subjected to CAT activity staining after native-PAGE separation.

### 4.3. Induced Resistance Bioassays

One day after treatment of either DW or SA (5 mM) on the leaves, the seedlings were subsequently sprayed with 1 × 10^5^ zoospores mL^−1^ of *P. palmivora*. The inoculated seedlings were then maintained in a climate-controlled room. Then, the disease evolution was evaluated for 4 days after the *P. palmivora* inoculation. The treated leaves at 4 dpi were collected for assays. Disease severity (DS) was recorded using a scale of 1–4, where 0 = no disease symptom, 1 = less than 10%, 2 = 11–30%, 3 = 31–50% and 4 = more than 50% of the leaf area appearing lesions [30]. It was expressed as a percentage (%) calculated according to the equation of Kranz [72].

DS (%) = {Σ(number of symptomatic plants × severity scale)] × 100}/(*N* × *Z*) = {[Σ(*n* × 0) + (*n* × 1) + (*n* × 2) + (*n* × 3) + (*n* × 4)] ×100}/(*N* × *Z*)

where, *N* = Total number of sampled plants; *Z* = Highest rating scale; *n* = number of symptomatic plants.

### 4.4. Protein Extraction, H_2_O_2_ Content and Enzyme Activity Assays

Leaf samples (0.5 g fresh weight) were frozen immediately in liquid nitrogen, ground to fine powder with a chilled mortar and pestle and then homogenized with 1 mL of cold 0.1 M Tris-HCl buffer, pH 7.0 containing 0.25% (*v*/*v*) triton-X and 3% (*w*/*v*) polyvinylpolypyrrolidone (PVPP). The extracts were then centrifuged at 12,000 rpm for 20 min at 4 °C. The supernatant was used for determining H_2_O_2_ content, enzyme activities and total protein content.

H_2_O_2_ content was measured by monitoring the reaction of undecomposed H_2_O_2_ with ammonium molybdate to generate a yellowish color, following the method of Hadwan and Abed [73]. The reaction mixture contained 200 μL of 50 mM sodium-potassium phosphate (Na_2_KPO_4_) buffer, pH 7.4 and 20 μL of enzyme extract and then incubated at 37 °C for 3 min. The reaction was terminated by adding 800 μL of 64.8 mM ammonium molybdate and then centrifuged at 12,000 rpm for 10 min. The absorbance of the obtained supernatant was recorded at 415 nm. The absorbance values were calibrated against an H_2_O_2_ standard curve and expressed as μmole per gram fresh weight (μmole g^−1^ FW).

CAT activity was determined by measuring the initial rate of H_2_O_2_ disappearance according to the method of Hadwan and Abed [73], with slight modifications. The reaction mixture contained 20 μL of enzyme extract and 200 μL of 100 mM H_2_O_2_ in 50 mM sodium-potassium phosphate (Na_2_KPO_4_) buffer, pH 7.4. The reaction was incubated at 37 °C for 3 min and then stopped by adding 800 μL of 64.8 mM ammonium molybdate. The decrease in H_2_O_2_ was followed by a decline in absorbance at 415 nm. One unit of CAT activity was defined as 1 μmole of H_2_O_2_ used in 1 min. The activity was represented as units per gram fresh weight (U g^−1^ FW).

POD activity was assayed following the method of Liu et al. [74] with slight modifications. The reaction contained 34 μL of enzyme extract, 33 μL of 0.25% (*v*/*v*) guaiacol, 33 μL of 0.1 M H_2_O_2_ and 900 μL of 10 mM phosphate buffer, pH 7.0. The reaction was assayed by monitoring the increase in absorbance at 470 nm. One unit of POD activity was defined as the amount of enzyme that provided a change of 0.01 in absorbance per min per gram fresh weight (U g^−1^ FW).

PAL activity was measured according to the method of D’ Cunha et al. [75], with some modifications. The reaction was started by addition of enzyme extract (70 μL) to 50 mM Tris-HCl pH 8.9 containing 1 mM β-mercaptoethanol and 0.1 M L-phenylalanine then incubated at 37 °C for 1 h. The reaction was stopped by adding 160 μL of 6N HCl and then centrifuged at 12,000 rpm for 10 min. The obtained supernatant was monitored by absorbance at 290 nm. One unit was defined as the amount of enzyme that caused an increase in absorbance for 0.01. PAL activity was identified as units per gram fresh weight (U g^−1^ FW).

Protein content was analyzed by the method of Bradford [76] using bovine serum albumin (BSA; Sigma Chem. Co., St. Louis, MO, USA) as a standard protein.

### 4.5. Histochemical detection of H_2_O_2_

In situ detection of H_2_O_2_ content in rubber tree leaf cells was performed by staining with 3, 3′-diaminobenzidine (DAB; Sigma Chem. Co., St. Louis, MO, USA) solution, according to the method of Thordal-Christensen et al. [77] with some modifications. Leaf samples were incubated in DAB solution (1 mg/mL; 10 mg DAB in 10 mL of 0.01 M phosphate buffer saline (PBS), pH 7.2) for 12 h under the dark and then boiled in 95% (*v*/*v*) ethanol for 10 min. H_2_O_2_ was visualized as a dark brown spot under a light microscope.

### 4.6. CAT Activity Staining after Native Polyacrylamide Gel Electrophoresis (Native-PAGE)

Crude protein extract (5 μL per lane) was electrophoresed on a 10% polyacrylamide gel without sodium dodecyl sulfate (SDS) under non-denaturing condition [78] for 4 h at 100 V. CAT bands were visualized by activity staining, according to the procedure of Woodbury et al. [79]. Briefly, after electrophoresis, the gel was placed in the dark and soaked in 1.3 mM H_2_O_2_ for 25 min. After that, the solution was poured out and the mixed solution in 1:1 ratio of 1% (*w*/*v*) K_3_Fe (CN)_6_ and 1% (*w*/*v*) FeCl_3_ was then applied on the gel. The gel was incubated with gentle shaking for 4 min and then rinsed with distilled water. Areas corresponding to CAT activity were visualized as yellow to light-green bands against a dark-green background on the gel because of the depletion of H_2_O_2_ by enzyme CAT.

### 4.7. Total Phenolic Content and Lignin Detection

Leaf samples (0.2 g fresh weight) were frozen immediately in liquid nitrogen, ground to a fine powder with a chilled mortar and pestle and then homogenized with 1 mL of sterile distilled water. The homogenate was centrifuged at 12,000 rpm for 20 min at room temperature. Total phenolic content of the extract was determined by the method of Torres et al. [80]. In brief, the supernatant 50 μL was mixed with 0.5 mL of 1 N Folin-Ciocalteu’s phenol reagent, and then incubated with gentle shaking at room temperature for 5 min. The mixture was combined with 1 mL of 20% (*w*/*v*) Na_2_CO_3_ solution, allowed to stand for 10 min and the absorbance was measured at 730 nm. Total phenolic content determined by a calibration curve of gallic acid was expressed as mg Gallic acid equivalents (GAE).

Lignin deposition was determined following the method of Jensen [81]. The leaves were soaked in 2% (*w*/*v*) phloroglucinol containing 20% (*v*/*v*) HCl for 20 min. The accumulation of lignin was visualized as red color spots.

### 4.8. SA and Scp Measurements

The contents of SA and Scp in the leaf of rubber tree seedlings treated with 5 mM SA were measured by high performance liquid chromatography (HPLC; Agilent1100, Waldbronn, Germany). Leaf samples (0.5 g fresh weight) were ground to a fine powder with mortar and pestle and then homogenized with 1 mL of 90% methanol, according to the method of Ederli et al. [82]. The homogenate was vortexed vigorously and then centrifuged at 12,000 rpm for 10 min at 4 °C. The supernatant was adjusted with 50% (*w*/*v*) trichloroacetic acid (TCA) to produce a final concentration of 5% (*w*/*v*) TCA, and subsequently filtrated through a 0.2 μm cellulose acetate fiber membrane. The chromatographic separation was achieved on a C18 reverse-phase column (ZORBAX Eclipse XDB-C18, 250 mm × 4.6 mm; 5 μm). The compounds in the sample (20 μL) were separated with mobile phase containing acetonitrile (ACN) and 0.1% (*v*/*v*) formic acid. The gradient condition was programmed as follows (time in min/percentage acetonitrile): 0–2/80, 8.5–10/60, 12/55, 13/40 and 15/15 with a flow rate of 1 mL min^−1^ under a controlled temperature column at 40 °C. The compounds from the separation were identified by a fluorescence detector with an excitation wavelength of 294 nm and emission wavelength of 426 nm for detecting SA and an excitation wavelength of 337 nm and an emission wavelength of 425 nm for detecting Scp. Each sample was conducted by HPLC with three independent replicates.

### 4.9. Total RNA Isolation and cDNA Synthesis

Rubber tree samples (0.2 g of leaves, stems, hypocotyls and roots of in-vitro rubber tree plantlets as well as leaves of rubber tree seedlings) were flash-frozen in liquid nitrogen and subsequently ground to a fine powder with a pre-chilled mortar and pestle. RNA isolation was done using the RNeasy^®^ Plant Mini Kit (Qiagen, Valencia, CA, USA), according to the manufacturer’s guidelines. The contaminating DNA was eliminated during total RNA purification step using an on-column RNase-free DNaseI digestion set (Qiagen, Valencia, CA, USA), according to the procedure of the RNeasy^®^ Plant Mini Kit. The RNA samples were measured for the quality and quantity by measuring a ratio of 260/280 nm absorption and their integrity was evaluated by visualizing the bands on a 1% agarose gel electrophoresis. Total RNA (2 μg) was reverse-transcribed into cDNA using the SuperScript^TM^ III Reverse Transcriptase (Invitrogen, Carlsbad, CA, USA), according to the manufacturer’s guidelines. The remaining RNA was eliminated from the cDNA products with RNase H (Invitrogen, Carlsbad, CA, USA). The first-strand cDNA was stored at −20 °C until use.

### 4.10. Isolation of Partial cDNAs of HbCAT and HbPAL

Amplification of *H. brasiliensis catalase* (*HbCAT*) was performed on first-strand cDNA templates of leaves, stems, hypocotyls and roots of in-vitro rubber tree plantlets using degenerate primers, CAT-F and CAT-R, (Table 1). Degenerate primers for *HbCAT* were designed based on the highly conserved sequences of two available published sequences of catalase genes from *H. brasiliensis*, (GenBank accession no. AAG43363 and ADU56199), in GenBank.

Amplification of *H. brasiliensis phenylalanine ammonia lyase* (*HbPAL*) was performed on first-strand cDNA templates of rubber tree seedling leaves using degenerate primers, PAL-F and PAL-R, (Table 1). Degenerate primers for *HbPAL* were designed based on the conserved sequences of the available published sequences of *PAL* genes from seven plants, *Manihot esculenta* (GenBank accession no. XM_021764705.1), *Ricinus communis* (XM_002519475.2), *Jatropha curcas* (XM_012226986.2), *Rhizophora mangle* (AY860427.2), *Durio zibethinus* (XM_022888871.1), *Theobroma cacao* (XM_007027292.2) and *Garcinia mangostana* (FJ197127.1), in GenBank.

The PCR reaction contained 7.5 μL of Emerald Amp^®^ GT PCR Master Mix (Takara, Otsu, Shiga, Japan), 0.67 μM of each degenerate primer and 0.5 μL of the first-strand cDNA (~100 ng). RT-PCR was done with a thermal cycler (TECHNE; TC-512 model), under the following conditions: preheating step at 94 °C for 1 min, 35 cycles of denaturing step at 94 °C for 1 min, annealing step at 60 °C for 1 min, extension step at 72 °C for 1 min, and a final elongation step at 72 °C for 10 min. The amplicons were analyzed by electrophoresis on 1.5% (*w*/*v*) agarose gel, visualized under the UV transilluminator and photographed by a Gel Documentary. PCR products of the expected size were gel-purified using the Gel/PCR DNA Fragments Extraction Kit (Geneaid, New Taipei City, Taiwan), ligated into pGEM^®^-T Easy Vector (Promega, Madison, WI, USA) and then transformed into *Escherichia coli* JM109 competent cells (Promega, Madison, WI, USA) by heat shock method. The transformants were selected on MacConkey agar plates containing 50 μg mL^−1^ carbenicillin. The recombinant plasmid was purified from 3 mL of bacterial culture using the E.Z.N.A.^®^ Plasmid Mini Kit I (OMEGA, Bio-Tek, Norcross, GA, USA) and subjected to sequencing by the Macrogen DNA sequencing service (Seoul, Korea).

The verified sequences were compared with other databases in GenBank via the basic alignment search tool (BLAST; https://blast.ncbi.nlm.nih.gov/Blast.cgi). The multiple sequence alignments were conducted using Clustal Omega (https://www.ebi.ac.uk/Tools/msa/clustalo/). Protein translation was performed using the Translate tool (ExPAsy; https://web.expasy.org/translate/).

### 4.11. Gene Expression Analyses of HbCAT1, HbPR1 and HbPAL By Semi-Quantitative Reverse Transcription Polymerase Chain Reaction (Semi-qRT-PCR)

The change of transcription levels of *HbCAT1*, *HbPR1* and *HbPAL* genes was evaluated by semi-qRT-PCR and the expression of *H. brasiliensis mitosis protein YLS8* gene (*HbMitosis*), a constitutively expressed housekeeping gene, was examined as an internal control [83]. The specific primers for *HbCAT1*, *HbPR1* and *HbPAL* genes were designed based on *HbCAT1*, *HbPR1*, and *HbPAL* genes, respectively. The PCR reaction was carried out using 10 μL of Emerald Amp^®^ GT PCR Master Mix (Takara, Otsu, Shiga, Japan), 0.25 μM of each specific primer (Table 2) and 0.5 μL of cDNA template (~100 ng). The semi-qRT-PCR reaction conditions were performed with an initial denaturation step at 94 °C for 4 min, followed by 25 cycles (for *HbCAT1* and *HbPR1* genes) and 35 cycles (for *HbPAL*) at 94 °C for 1 min, annealing step at 60 °C for 1 min, extension step at 72 °C for 1 min and a final elongation step at 72 °C for 10 min. The PCR products were analyzed by electrophoresis on 1.5% agarose gel, visualized under the UV transilluminator and photographed by a Gel Documentary. The band intensity on the gel was measured by the Image-Using VisionWorksLS software (UVP BioSpectrum^®^ MultiSpectral Imaging System^TM^, Cambridge, UK).

### 4.12. Statistical analysis

All the experiments were conducted using 10 plants per replicate and each treatment was replicated three times. A *p*-value ≤ 0.05 indicated a significant difference, and the data was presented as the mean of three independent experiments with similar results. All data were subjected to one-way analysis of variance (ANOVA) followed by Tukey’s HSD test using SPSS Statistics software, version 17.0. The data of disease severity index (DSI) were analyzed using the paired-samples *t*-test.

## 5. Conclusions

The present study suggested that exogenous SA application could effectively enhance resistance against *P. palmivora*-infected rubber tree. SA could induce the coordinate activation of defense responses, such as the accumulation of H_2_O_2_, the induction of defense-related enzymes activities (CAT, POD and PAL), endogenous SA, Scp, and lignin deposition, including the up-regulated expressions of *HbCAT1*, *HbPAL* and *HbPR1* genes. In summary, our findings provide valuable information regarding the role of SA enhancing tolerance in rubber tree to *P. palmivora* infection and the underlying defense mechanisms.

## Figures and Tables

**Table 1:** Degenerate primers for isolation of *HbCAT* and *HbPAL* genes.

Primer Names	Sequence (5′→3′)	Tm (°C)	Base Pairs (bp)
*CAT*-F	ACWTGTGCTGATTTCCTTCGAG	55.0–60.3	22
*CAT*-R	ATGGTGATTGTTGTGATGAGCACAC	58.6–64.2	25
*PAL*-F	CATTTGGATGARGTGAARARAATGGT	51.7–56.4	26
*PAL*-R	AGTTSACRTCTTGGTTGTGTTGCTC	56.0–57.7	25

**Table 2:** Specific primers for semi-qRT-PCR.

Genes	Primer Names	Sequence (5′→3′)	Amplicon Size (bp)	GenBank Accession No.
*HbCAT1*	*HbCAT1*-F	CCTGTCATTGTCCGTTTCTCCACTG	495	MF383167
	*HbCAT1*-R	CCAGCGGCAATGGAGTCATATAAATCC		
*HbPAL*	*HbPAL*-F	TGAACGAGGGAACTGATAGCTATGGTG	736	MG992015
	*HbPAL*-R	GGTTTCTGCAAGGGATCCATTTCATGC		
*HbPR1*	*HbPR1*-F	ATGCCCATAACCAAGCACGAGCAG	364	KM514666
	*HbPR1*-R	CCAGGAGGGTCGTAGTTGCATCCA		
*HbMitosis*	*HbMito*-F	TGGGCTGTTGATCAGGCAATCTTGGC	577	HQ323250
	*HbMito*-R	TGTCAGATACATTGCTGCACACAAGGC		
